# Mesenteric Paraganglioma: An Unusual Suspect To Consider

**DOI:** 10.7759/cureus.71021

**Published:** 2024-10-07

**Authors:** Andreia Martins Fernandes, Raquel Prata Saraiva, Leandro Augusto Silva, Sara Reis, Joana Couto

**Affiliations:** 1 Endocrinology, Portuguese Institute of Oncology of Coimbra, Coimbra, PRT; 2 Surgery, Portuguese Institute of Oncology of Coimbra, Coimbra, PRT; 3 Pathology, Portuguese Institute of Oncology of Coimbra, Coimbra, PRT

**Keywords:** abdominal neoplasms, extra-adrenal paraganglioma, mesentery, neuroendocrine tumors, paraganglioma

## Abstract

Mesenteric paragangliomas (PGLs) are extremely rare, with a limited number of reported cases. These tumors are typically non-functioning and commonly manifest as a palpable abdominal mass or abdominal pain; however, a significant proportion of patients remain asymptomatic. Despite their rarity, they should be considered in the differential diagnosis of mesenteric masses. Surgical treatment is the preferred approach, and even after successful complete resection, long-term follow-up is crucial due to the unpredictable potential for metastasis.

In this report, we describe the case of a 48-year-old male patient who presented with a self-limited episode of severe abdominal pain, without hyperfunctioning symptoms. The physical examination was unremarkable. Imaging studies revealed the presence of a mesenteric mass. The clinical, laboratory, imaging, and biopsy findings were insufficient to differentiate between an epithelial neuroendocrine tumor and a paraganglioma. Following a review by a neuroendocrine multidisciplinary team, surgery was proposed. While the initial suspicion was an epithelial neuroendocrine tumor, the histopathological examination of the surgical specimen was consistent with a PGL. Currently, after two years of follow-up, the patient remains asymptomatic and is undergoing regular clinical monitoring.

## Introduction

Paragangliomas (PGL) are rare tumors, with an estimated overall incidence of 1/300,000 [1]. These tumors are derived from paraganglia and can develop in either the parasympathetic or sympathetic nervous system [1]. Parasympathetic PGLs arise from paraganglion cells of the autonomic nervous system, almost exclusively confined to the head and neck region. In contrast, sympathetic PGLs develop along the sympathetic chain, spanning from the skull base to the bladder, with a higher prevalence in the abdomen [1, 2]. The mesentery is an exceedingly rare location for these tumors, with only 36 cases reported in the literature [3,4]. PGLs can be classified as functioning or nonfunctioning based on their capacity to secrete catecholamines. Among the functioning types, PGLs derived from the adrenal medulla are the most common, accounting for 80-85% of cases, followed by extra-adrenal abdominal PGLs [1].

We report a rare case of a nonfunctional PGL arising from the mesentery. The patient experienced a self-limited episode of severe abdominal pain, without any other symptoms, and imaging examinations revealed a mass in the mesentery. Although the initial suspicion was an epithelial neuroendocrine tumor, the histopathological examination of the surgical specimen was consistent with a mesenteric PGL.

## Case presentation

**Figure 1:**
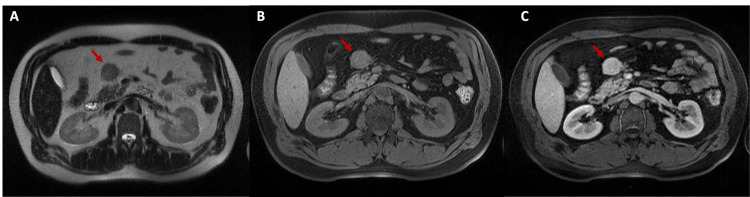
MRI scanning results. A) MRI T2 HASTE axial image showing a T2 hypointense mesenteric mass. B) MRI T1 fat-saturated pre-contrast axial image showing a T1 isointense mesenteric mass, indicating an absence of a fat component. C) MRI T1 fat-saturated post-contrast axial image showing enhancement in the mesenteric mass, suggesting hypervascularity.

A 48-year-old man with a history of asthma and a previous isolated substance-induced psychotic episode presented with a self-limited episode of severe abdominal pain. He denied any other symptoms. His family history was unremarkable. An abdominal ultrasound was performed and revealed a mass apparently associated with the pancreas. Subsequent unenhanced abdominal CT showed a well-defined, slightly lobulated solid mass measuring 34.6 x 28.4 mm, located within the mesenteric fat and contiguous with the anterior contour of the pancreatic head/body transition. MRI revealed a well-defined hypervascular mass measuring 35 x 31 mm in the mesentery, located anterior to the cephalic region of the pancreas, with a discernible separation from it (Figure 1).

Following these imaging findings, the patient was promptly referred to our cancer centre.

Physical examination showed the patient in good general status, with unremarkable abdominal palpation. An ultrasound-guided biopsy revealed the presence of a well-differentiated neuroendocrine tumor with a Ki-67 proliferation index of less than 1%. Whole-body positron emission tomography (PET)/CT Gallium-68-labeled 1,4,7,10-tetraazacyclododecane-N,N',N'',N'''-tetraacetic acid-NOC (68Ga-DOTA-NOC) supported the diagnosis of a neuroendocrine tumor of the mesentery and did not identify any other abnormal uptakes. Analytical tests showed no elevation of chromogranin A (CgA), neuron-specific enolase (NSE), pancreatic polypeptide, gastrin, urinary 5-hydroxyindoleacetic acid (5-HIAA), serum calcium, parathyroid hormone, or plasma metanephrines (Table 1).

**Table 1:** Laboratory findings of the patient.

Laboratory parameters	Result	Units	Reference range
Ionized Calcium	1.15	mmol/L	1.14-1.29
Parathyroid Hormone	85	pg/mL	18-80
Chromogranin A	94.7	ng/L	≤102
Neuron-Specific Enolase	9.5	ng/mL	≤20
Pancreatic Polypeptide	12.1	pmol/L	≤100
Gastrin	13.8	pg/mL	13-115
Plasma Metanephrine	56.8	pg/mL	≤65
Plasma Normetanephrine	87.1	pg/mL	≤196
Plasma 3-Methoxytyramine	93	pg/mL	≤175
Urinary 5-Hydroxyindoleacetic Acid	3.9	mg/24h	≤15

**Figure 2:**
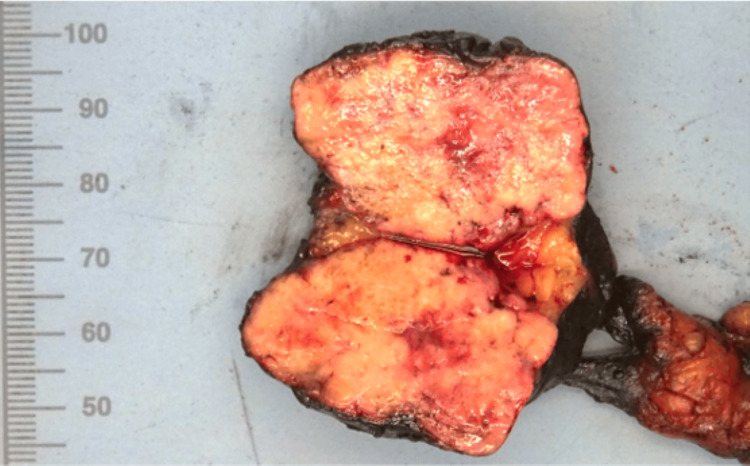
Macroscopic view of the surgical specimen.

Following a review by a neuroendocrine multidisciplinary team, surgery was proposed. Intraoperatively, the mass was located at the root of the mesentery, close to the inferior border of the pancreas, and had no involvement with adjacent structures, namely the superior mesenteric vessels. The tumor was completely resected with grossly free margins, without necessitating small bowel resection. No other lesion was found during the small bowel inspection. The surgery and postoperative period were complication-free. Macroscopic examination found a mass measuring 47 x 40 mm with well-defined margins and without encapsulation (Figure 2).

**Figure 3:**
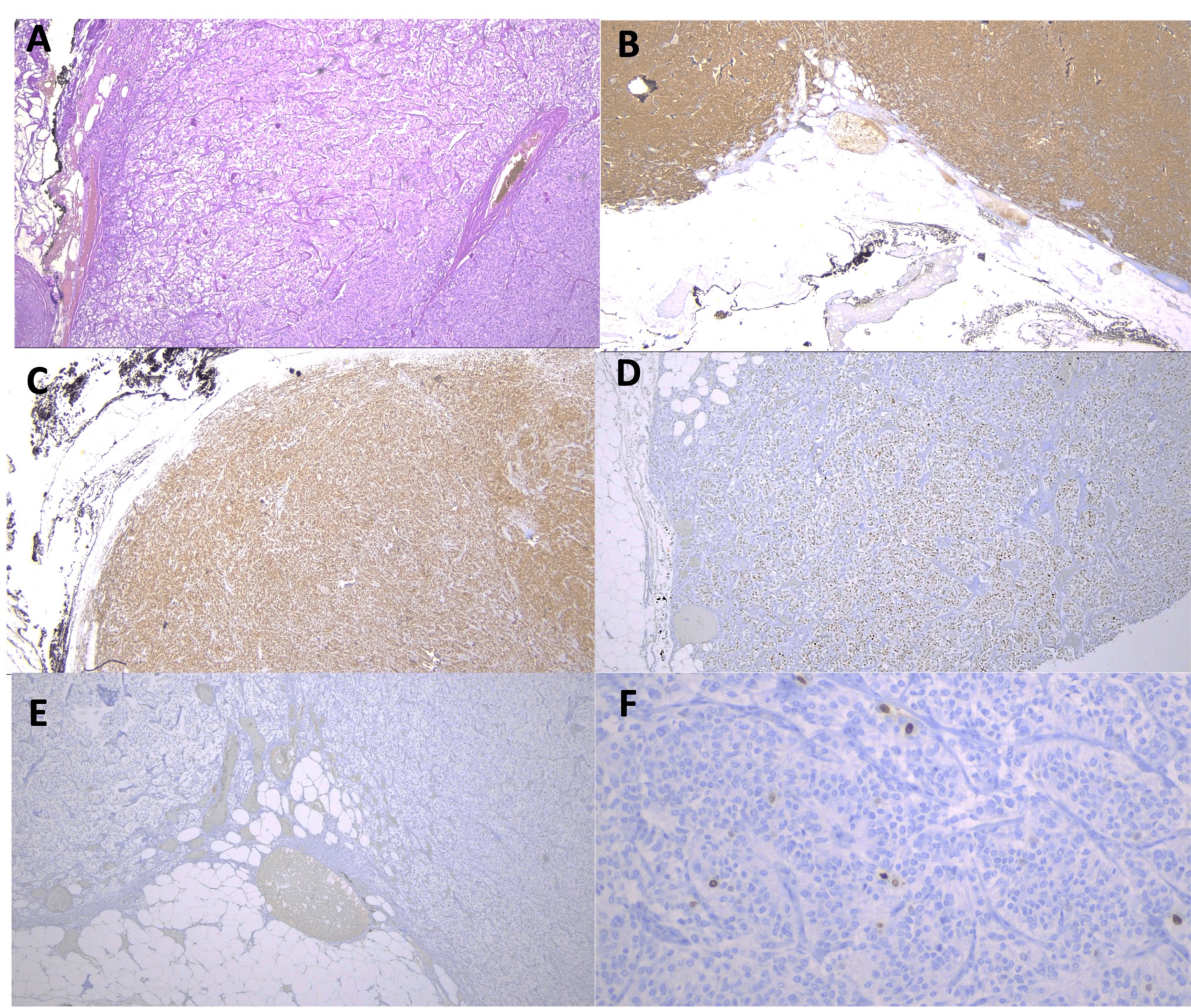
Histological examination. A) 4x magnification of a H&E stain displaying the characteristic nesting pattern (Zellballen) of tumor cells, arranged in nests and trabeculae, separated by fibrovascular connective tissue. B, C) 2x magnification with immunohistochemistry (IHC) staining for chromogranin A and SDHB, respectively. D) 4x magnification with IHC staining for GATA3. E) 4x magnification showing no IHC staining for keratins AE1/AE3. F) 20x magnification with Ki-67 immunostaining, indicating a low proliferative rate of 1-2%.

Histological examination revealed tumor cells arranged in nests and trabeculae, separated by a thin fibrovascular stroma, exhibiting a characteristic Zellballen pattern. There were no abnormal mitotic figures, necrosis, or evidence of lymphovascular invasion. Immunohistochemistry (IHC) showed positive staining for chromogranin A, S100 protein, CD56, synaptophysin, GATA3, and SDHB, while staining was negative for cytokeratin 8/18 (CK8/18), AE1/AE3, glial fibrillary acidic protein (GFAP), carbonic anhydrase IX (CAIX), and alpha-inhibin. The Ki-67 proliferation index was 1-2% (Figure 3).

These findings were consistent with a diagnosis of mesenteric PGL. Considering the non-functional biochemical phenotype, histological parameters, and Ki-67 proliferative index, the PGL was classified as moderately differentiated based on a total Grading of Adrenal Phaeochromocytoma and Paraganglioma (GAPP) score of 3, indicating an intermediate risk of metastasis.

Following the diagnosis, the patient underwent genetic testing, which identified no pathogenic or likely pathogenic variants, nor duplications or deletions, in the following genes: CDKN1B, EGLN1, FH, GDNF, KIF1B, MAX, MEN1, NF1, PRKAR1A, RET, SDHA, SDHAF2, SDHB, SDHD, TMEM127, and VHL.

Currently, two years after surgery, the patient remains asymptomatic and is undergoing regular clinical monitoring, including abdominal-pelvic MRI and laboratory tests.

## Discussion

Extra-adrenal PGL can potentially arise in various locations where paraganglia are typically found. In the abdominal region, PGL primarily arise from retroperitoneal paraganglia, commonly found in the para-aortic area. Mesenteric PGL are exceptionally rare, with only 36 cases reported in the literature [3]. There is a hypothesis proposing that these tumors originate from mesenteric paraganglia, which develop as a result of the migration of paraganglionic cells along the root of the superior mesenteric artery. Due to this location, mesenteric PGL present potentially high morbidity if isolation from the mesentery is unsuccessful and small bowel mesentery resection is needed [5, 6].

Due to the low incidence of reported cases, there is limited understanding regarding the pathogenesis of these PGL, which can occur either sporadically or be inherited [7]. Up to 40% of patients have germline mutations, and somatic mutations can be found in an additional 30-40% of patients with sporadic PGL [7, 8]. The majority of hereditary PGL cases are associated with germline mutations in the subunits of succinate dehydrogenase (SDH) genes [9]. Therefore, genetic testing is recommended in all patients diagnosed with PGL, playing a crucial role in patient management, influencing both treatment choices and clinical follow-up [8, 10].

Mesenteric PGL primarily affect female patients, with an average age of 57 years [3]. Typically non-functional, these tumors commonly manifest with a palpable abdominal mass or nonspecific abdominal pain; however, a significant proportion of patients remain asymptomatic. In cases where patients exhibit no symptoms, they are often discovered incidentally during imaging examinations performed for unrelated reasons [3]. In rare cases, these tumors can be functional, resulting in the production of significant levels of catecholamines, and they may manifest with paroxysmal hypertension, along with the classical triad of palpitations, headaches, and profuse sweating [7]. The preoperative diagnosis of extra-adrenal PGL can be challenging, especially in cases where the tumor arises from uncommon sites or when symptoms related to excessive catecholamine levels are absent, as in the current case [11].

A definitive diagnosis requires histopathologic examination of the tissue. However, if PGL is suspected, it is not recommended to perform a biopsy before ruling out a catecholamine-secreting PGL, as this procedure carries the risk of triggering a catecholamine crisis in the absence of proper alpha and beta blockade [12].

Nonetheless, in our case, a biopsy of the mass was conducted because there was no clinical, laboratory, or imaging suspicion of that diagnosis at this point. The preoperative study did not allow for the distinction between a primary epithelial neuroendocrine tumor of the mesentery, a mesenteric metastasis from a small bowel neuroendocrine tumor, and a mesenteric PGL. Similar to PGL, primary neuroendocrine tumors of the mesentery are extremely rare. In contrast, midgut neuroendocrine tumors often spread to the mesentery [13].

PGL and neuroendocrine tumors from different organs can share similarities in their nested structure and expression of neuroendocrine markers such as chromogranin A and synaptophysin. This similarity can lead to diagnostic challenges. However, the absence of keratin expression in PGL is a useful feature for distinguishing them from most neuroendocrine tumors. Also, GATA3 expression is typically found in the majority of PGL but not in neuroendocrine tumors, making it a valuable marker for differentiation. While S100 protein-positive cells are commonly seen in PGL, they can also be present in certain neuroendocrine tumors [14]. In our case, the tumoral cells demonstrated positive staining for synaptophysin, chromogranin A, S100 protein, and GATA3, while showing no expression of keratins AE1/AE3 or CK8/18. These immunohistochemical findings confirmed the diagnosis of PGL. Based on the patient's history and relevant examination results, the final diagnosis was a non-functional mesenteric PGL.

Given the absence of definitive characteristics that can predict metastatic behavior, it is widely recognized that all PGL have the potential to metastasize [15]. The current assessment of PGL metastatic potential relies on a comprehensive evaluation of several factors, including tumor size (≥5 cm), the presence of an SDHB mutation, a dopaminergic phenotype, and a high Ki-67 index. Furthermore, for a more precise risk assessment, it should incorporate the Pheochromocytoma of the Adrenal Gland Score (PASS) and the GAPP score, which are the only globally used risk-stratification systems based on histological features [16].

The treatment requires a multidisciplinary team and involves surgical removal of the tumor [10]. PGL located in the small bowel mesentery frequently require resection of adjacent bowel because of their proximity to the bowel or the risk of devascularization [5]. In the current case, however, the mesenteric mass did not invade any vascular structures. Consequently, surgical morbidity was minimized, and organ resection was not necessary.

There are no cases of tumor recurrence described in the literature [3, 5]. Taking into consideration the potential for metastasis, in addition to the rarity of this pathology and the limited knowledge surrounding it, subsequent long-term follow-up of all these patients is indicated [4, 16]. Currently, there are no specific follow-up protocols established for mesenteric PGL. Whenever possible, follow-up should be managed by a multidisciplinary team at a tertiary care center. Overall, it is recommended to perform annual biochemical testing (including plasma or urinary metanephrines and 3-methoxytyramine [3-MT], and CgA in metanephrine/3-MT-negative PGL) combined with imaging studies every 1-2 years, for early detection of tumor recurrence or metastatic disease. To minimize cumulative radiation exposure, MRI is preferred as the primary imaging method for surveillance [17].

## Conclusions

In summary, the evaluation of a mesenteric mass can be challenging. In this case, clinical, laboratory, imaging, and biopsy findings were insufficient to distinguish between an epithelial neuroendocrine tumor and PGL. Therefore, a definitive diagnosis was only possible after examining the surgical specimen. Mesenteric PGL are often non-functioning, as seen in this case, and, although rare, should be included in the differential diagnosis of solid hypervascular mesenteric masses due to their potential to secrete catecholamines. This secretion can lead to severe outcomes if not properly managed, as well as their malignant potential and possible association with genetic syndromes. Surgical resection is the treatment of choice, and even after a successful complete resection, it is important to provide long-term follow-up.
